# Imagining the Unimaginable: Parricide in Early Modern England and Wales, c.1600–c.1760

**DOI:** 10.1177/0363199016644706

**Published:** 2016-05-17

**Authors:** Garthine Walker

**Affiliations:** 1 Department of History, School of History, Archaeology and Religion, Cardiff University, Cardiff, UK

**Keywords:** parricide, insanity, homicide, parental cruelty, criminal trials, gender, age, violence

## Abstract

This article explores the ways in which parricide was comprehended in England and Wales, c.1600–1760, and shows that while some parallels exist with modern explanatory models of parricide offenders, they had very different meanings in the early modern context. While both lunacy and the cruelty of parents were understood as possible contexts for parricide, neither were common. The dominant explanation was the gratuitous violence of a selfish individual who lacked compassion and who saw the parent as an obstacle—to an inheritance, riches, marriage, and freedom—to be removed. The article explores these three categories and suggests ways in which this began to change in the mid-eighteenth century.

Parricide was viewed an atrocious crime in early modern Europe.^1^ In many countries, the murder of a father (patricide) or mother (matricide) by their son or daughter was a special form of aggravated homicide. In England and Wales, however, parricide was not a distinct common law or statutory offense; it was prosecuted under regular homicide law, where it constituted a small proportion of cases that came before the courts. Parricide nonetheless enjoyed a cultural notoriety. Political commentaries on regicide described “pa[t]ricide, matricide, and fratricide” as “the three blackest” of crimes.^2^ Popular trial pamphlets and execution ballads asserted that of all “horrid variety of bloody murders” none was “more barbarous, unnatural, and detestable.”^3^ Parricide was “a crime of so black a dye” that a “vast concourse of people” inevitably turned out to witness “the trial and catastrophe of so execrable an offender.”^4^ What kind of person, one might ask, could commit such an act?

Historians of early modern England and Wales have been surprisingly quiet on the subject. Violence perpetrated by children against parents has rarely been addressed within historiographies of the early modern family, crime, or women and gender. The vast body of work on domestic violence has focused primarily on that between husbands and wives and, to a lesser degree, upon that inflicted by parents, masters, and mistresses upon children and servants and upon neonatal infanticide. The neglect of parricide reflects the origins and evolution of these historiographies and the nature of the primary evidence. The history of the early modern English family developed partly as a response to pessimistic views of affective relations published in the 1960s and 1970s by Lawrence Stone and others and thereafter emphasized positive emotional attachments of family members.^5^ Neither have recent studies of parenting and parent–child relations within the history of emotions interrogated violence against parents.^6^ The social history of early modern crime was dominated first by quantification in which parricide hardly figured as a category and then by the cultural turn toward popular texts such as pamphlets and ballads, where other forms of violence overshadowed parricide. Certain offenses with low prosecution rates have been selected for study by historians, but these have tended to be those that either produced significant subgenres of popular crime literature such as witchcraft and petty treason or were specific statutory offenses such as rape and the concealment of the deaths of newborn bastards (infanticide). Historians of women and gender, meanwhile, were initially most concerned with the ways in which patriarchy legitimated male violence toward women and children and invalidated responses to it by subordinate members of the household. Even sophisticated studies that have illuminated the early modern household as a site of conflict—indicated by their titles in the cases of Frances Dolan’s *Dangerous Familiars* and Laura Gowing’s *Domestic Dangers*—have left out violence against parents.^7^ All this research nonetheless provides us with a rich and detailed context in which to situate a study of early modern parricide.

Throughout the early modern period, parricide was condemned as so unnatural an act that it was scarcely comprehensible. In 1606, for instance, a pamphlet about a patricide–suicide stated that while “all killing is cruel and an enemy to nature, yet the unnatural and detested parricides … astonisheth all human reason to consider it, appalleth all the senses to apprehend it, and exceedeth all credulity to believe such a thing to be done, till they behold it actually performed.”^8^ A century and a half later, a daughter’s murder of her father was described as a “crime so shocking … and so aggravated in all its circumstances” that it will “make our children’s children, when they read the horrid tale of this day, blush to think that such an inhuman creature ever had an existence.”^9^ We can, however, do more than simply acknowledge the dismay and consternation with which contemporaries viewed parricide as “unnatural.” Even these murder pamphlets are not entirely commensurate. The first was a piece of anti-Catholic propaganda written by a Puritan preacher and published in the wake of the Gunpowder Plot. The second was an officially sanctioned trial transcript published with the Oxford Assize judges’ approval. It was one of scores of commentaries on the trial of one Mary Blandy that were produced in a media frenzy that would have been inconceivable a century and a half earlier and which dissected a complex tale that touched on the gentry marriage market, thwarted romance, clandestine marriage, secret love letters, illegitimate children, love potions, and poisoned tea and porridge.

In this article, I shall first consider the legal status of parricide and how legal treatises dealt with it before identifying and discussing three “types” of parricide that contemporaries evoked in their discussions of the crime, its perpetrators, and its treatment by the courts.^10^ I shall end by exploring tensions between these discrete categories in publicity surrounding the Blandy case. What follows is based on a range of primary evidence, especially murder trial pamphlets and execution ballads produced across the entire period, newspaper reports and printed accounts and transcripts of trials held at the provincial Assizes and at London’s Old Bailey from the later seventeenth century onward, and manuscript documents relating to such trials and the legal process.^11^ Such sources do not collectively provide a bucket into which we can simply dip to retrieve information about parricide. Each category of source had its own conventions and restrictions, and in some types of document, such as murder pamphlets, the depiction of crime may be seen to be as much in the service of the genre as vice versa. My approach to these materials seeks to be sensitive to particular types of evidence while identifying and analyzing behavioral characteristics of parricide and the ways in which early modern people made sense of it. At the center of my analysis are over forty parricides that I have identified in trial records, popular printed texts, and news reports between 1600 and the 1750s. I have not sought to determine the motives of offenders per se. Rather, I have been concerned with the motives that contemporaries attributed to perpetrators, the circumstances within which they perceived parricide to occur, and the ways in which they ascribed culpability for the crime. Identifying early modern interpretative categories allows us to historicize an otherwise seemingly transhistorical phenomenon.

## An Unimaginable Crime

In early modern England and Wales, where theories of social order were modeled on the “natural” hierarchies of the family and household, parricide was a cultural category despite not having the status of a discrete criminal offense. Anyone with a legal education knew that under Roman law, parricide “was punished in a much severer manner than any other kind of homicide.”^12^ Nonetheless, news from abroad made clear that in practice parricide was usually punished like any other heinous crime.^13^ Yet parricide posed a problem for English jurists. Logically, it should have been encompassed by the legal category of petty treason. Children, after all, were subject to and owed duty and obedience to parents who had civil sovereignty over them, just as wives were and did to husbands, and servants and apprentices to masters and mistresses. Legal manuals written for both jurists and laymen frequently referred to parricide in discussions of petty treason if only to explain its absence. Sir Edward Coke, one of the most senior judges in England, opined in 1628 that the treason statute omitted parricide because “the law-makers never imagined any child would do” such a thing. Blackstone made the same point in 1769.^14^ It was, effectively, an unimaginable crime. Only if the child “served the father or mother for wages, or meat, drink, or apparel,” and thus performed the role of a servant, did the offense come within the remit of the statute. Thus, Ferdinando Pulton noted that “the Treason should be in respect of the duty of service broken, and not of duty of nature violated.”^15^ This was not entirely satisfactory. As Coke conceded, “the offence is far more heinous and impious in a child than in a servant, for *peccata contra naturam sunt gravissima* [sins against nature are very serious].”^16^

Not everyone agreed. Some asserted that petty treason did encompass parricide.^17^ Michael Dalton’s *Countrey Justice* (1618) included premeditated parricide among a list of circumstances that constituted petty treason: Pulton’s view notwithstanding, “it is Treason in the child, in respect of the duty of nature violated.” If the victim was a stepparent “with whom they dwell and do service, and have meat and drink, it is [also] petty treason, although such child take no wages; but the indictment shall be by the name of servant.” This view was reiterated by others, including Sir Matthew Hale.^18^ The relationship between the wording and meaning of statutes was a perennial issue. It was, after all, an interpretative act to include mistresses killed by servants within petty treason, given that the Treason Act mentioned only masters.^19^ In the early nineteenth century, legal experts were still debating whether petty treason extended to children who killed parents.^20^ Nonetheless, all accepted that parricide was particularly dreadful. Convicted parricides were often hanged in chains at judges’ discretion,^21^ their bodies sent to be anatomized like those of other odious felons,^22^ and some did indeed suffer as petty traitors.^23^ At Tyburn, in the summer of 1735, where two of the four persons hanged were parricides, the “Mob were so sensible of the heinousness of the crimes these villains had been guilty of,” that “they pelted them with Dirt even when they were tied up to the gallows.”^24^

The maxim that historically lawmakers failed to legislate against parricide because it was unimaginable was endlessly repeated. It appeared not only in legal manuals and reports of actual parricides but in other contexts too. Discussions of apparently unrelated events such as the South Sea Bubble in the 1720s evoked the absence of ancient laws against parricide, “it never entering into [legislators’] minds that any could be guilty of so villainous a crime”; a crime “so monstrous and shocking, that wise States would not suffer [it] to stand in their Statute Books; because they would not put such an indignity upon human nature, as to suppose it is capable of committing them. They would not mention what they imagined would never be practiced.”^25^ Yet early modern people not only imagined parricide but did so in a variety of ways.

## Imagining the Unimaginable

Accounts of parricide were constructed within terms of legal and societal attitudes to lethal violence, to the relationship of perpetrator and victim, and to the particular dynamics of the incident in question. Legal culpability for homicide was evaluated according to a principle of finite culpability. When victims were held accountable for the immediate situation that had caused their death, the killer was deemed to be only partially responsible. The category of manslaughter, for example, acknowledged that the deceased had contributed to the incident in which they were killed, most commonly by provoking their assailant or voluntarily entering what turned out to be a lethal fight. Violence had also to be proportionate. Excessive violence in retaliation for a minor slight shifted a partially culpable homicide (manslaughter) into a fully culpable one (murder).^26^ Men and, from 1692, women convicted of manslaughter had a good chance of having their death sentence commuted to branding or, in the eighteenth century, to transportation. In contrast, the penalty for murder was death, with few ways of avoiding it.^27^ These general classifications of lethal violence took on particular meanings when incidents involved parents and children.

Modern criminologists have identified three types of parricide offender according to the perpetrator’s perceived motives: first, people suffering severe mental illness; second, those who kill in order to end severe and ongoing physical, emotional, and/or sexual abuse; and third, dangerously antisocial or psychopathic persons who, lacking empathy, kill parents for selfish reasons in the manner of removing an obstacle to something they want, such as money, an inheritance, or greater freedom.^28^ These categories cannot simply be imposed onto early modern parricides, of course: what constitutes mental illness, “abuse,” healthy parent–child relations, and empathy is historically and culturally variable.^29^ There are, nonetheless, interesting points of comparison. Just like us, early modern people differentiated parricidal killings in order to make sense of them. The three categories that I shall discuss are first, parricide enacted by a lunatic who knew not what he or she did; second, parricide perpetrated by a cold-blooded, cruel killer who lacked human feeling; and third, parricide that was the ultimate consequence of parental fault. These categories may look similar to those employed by many modern criminologists, but, as we shall see, they were not understood in the same way in the early modern context.

### Parricide and Insanity

Insanity had long been evoked as a potential means of understanding otherwise inexplicable behaviors. With regard to crime, early modern people were aware that someone’s state of mind was relevant to both motive and accountability. Although the formal insanity defense was established only in the nineteenth century,^30^ early modern juries could and did return verdicts of non compos mentis when they considered a defendant to have been incapable of understanding the nature of what they were doing and that it was wrong. Such a “defect of reason” had to be caused by a “disease of the mind,” which could not be claimed by all such defects. Being mentally incapacitated by alcohol consumption, for example, did not come into this category: while “reason departeth, when drink possesseth the brain,” it was a “voluntarily ignorance in him, in as much as such ignorance cometh to him by his own act and folly.”^31^ Technically, someone who was non compos mentis could not be convicted of felony as there could be no criminal intent (*mens rea*). The law recognized three classes of such persons: those who were born “fools” (idiots); those who lost their memory and mental faculties as a consequence of accident, illness or otherwise unfathomable reasons (in which case the cause was attributed to a visitation of God); and lunatics. Lunacy was regarded as a temporary state interspersed with periods of lucidity and was often associated with violent and threatening behaviors.^32^ It is in this context that people suggested lethal violence had been committed by lunatics, whether to explain a killer’s behavior or to mitigate it at trial: they did not speak of “lunatics” killing people in any modern colloquial sense of referring to someone who was excessively or inexplicably angry.

One might suppose that insanity would be commonly employed to explain an allegedly unimaginable crime such as parricide. Certain other familial homicides without easily discernible motives were associated with insanity in the eighteenth century, such as married parents who killed legitimate children in a temporary “fit of frenzy.”^33^ In such cases, defendants had usually to have been already identified as insane or have clearly displayed peculiar behavior that could be attributed to insanity, such as episodes of incoherent conversation and nonsensical actions.^34^ A minority of early modern parricides were indeed explained in these terms, as in the case of a Bedfordshire man, “for some time disorder’d in his Senses,” who in 1749 burnt his house and outbuildings to the ground and shot his mother, killing her on the second attempt.^35^ Similarly, a young fellow in Surrey was prosecuted for arson and attempted matricide in 1740, but “some Symptoms of Lunacy appearing in his Favour, the Court was pleased to dismiss him.”^36^

However, unlike modern assessments of adult offender parricides, which are often attributed to severe mental illnesses, only a few early modern parricides were explained—and even fewer excused by the courts—as acts of insanity.^37^ Take Mr. Bishop, for instance, a Lincolnshire maltster who in 1725 arose from his bed in the dead of night, went to his mother’s room and “cut her throat from ear to ear with a pen knife in a most barbarous manner.” He also stabbed, almost fatally, the gentleman lodger who had rushed upstairs in attempt to help the woman, “ripping up” his belly so that his “cawl hung out.” A maidservant and a young child who had been asleep in the same room, despite being “almost frighted to death,” managed to lock themselves in a garret and were rescued out of the window. Having committing these bloody acts, Bishop calmly returned to bed and was thence arrested without making resistance. During the following weeks, in Lincoln Gaol awaiting trial, he twice attempted suicide. Early news reports described him as a lunatic; indeed, his mother was present only because his wife had “sent for [her] in order to pacify him.” The coroner’s jury, however, brought in a verdict of willful murder, albeit “after several consultations,” after which “great Endeavours” were made “to prove him Lunatic [for the benefit of the trial jury] at the Assizes.”^38^ Historians of madness have perhaps overestimated the association between insanity and family violence because their sources have been those that discuss madness per se rather than domestic violence in particular. There is, in fact, little evidence to support the view that contemporaries believed “defiance towards elders and superiors within the family [w]as a sign of madness” or that disobedience toward parents “was itself a sign of mental abnormality.”^39^ The sixteenth-century physician, Richard Napier, included “will not be ruled” as a potential symptom of madness, yet it accounted for under 3 percent of his patients’ symptoms. Moreover, although Napier did not class a single one of his 158 suicidal patients as mad or a lunatic, jurors were far more likely to return verdicts of non compos mentis in cases of suicide than in any other form of homicide.^40^

When lunacy was presented as an explanation for lethal violence, it was evaluated by the court like any other evidence. Thus, in the case of Robert Hicks, tried for matricide in 1722, acquittal did not rest on the case he made for being insane. The evening before his mother died, she had locked him out of the house after he turned up “fuddled,” in response to which he broke her windows and shutters; later, they made up, but when he put “his hands about her neck” to kiss her goodnight, she had “shriek’d out … ‘My Rogue has Murder’d me!’” In his defense, Hicks claimed that he suffered from violent rages caused by an injury sustained in service to the crown, having “crack’d my scull on board the *Revenge*,” a royal navy battleship. Witnesses on his behalf asserted that “he was a Lunatic,” “quite mad and out of his senses when he gets drunk”; two months before, he had been “quite crazy, he had two nurses to look after him, and he still has relapses at the full of the moon.” Yet the Old Bailey *Proceedings* did not publish these statements, mentioning his alleged lunacy only briefly and in passing. His acquittal rested on other evidence. No one had seen Hicks strike his mother; she had complained of no hurt to the neighbors who sat with her until bedtime, who in any case described her as “a very spiteful woman” who frequently cried “‘murder!’ when nobody touch’d her.” Crucially, two surgeons found no bruise, mark, discoloration, or other sign that she had received a lethal blow the night before. She had died, they believed, of a “convulsion-fit,” probably brought on by her own “violent passion,” not her son’s.^41^

Lunacy was not the default or most common explanation for parricide but only one possibility. Whereas the modern tabloid press likes to sensationalize lethal violence committed by those suffering from psychiatric conditions, in the seventeenth and eighteenth centuries, murders committed by those non compos mentis were deemed *less* sensational than those who had no such mitigating circumstances. There were nonetheless contexts in which insanity was presented in popular print in sensationalist terms, notably when loss of reason was associated with extreme religious zeal. Depicting religious “deviants” or “extremists” as capable of treachery and murder was a popular literary trope. A pamphlet of 1606 described a brutal parricide committed in “the frantic spirit of a Papist.”^42^ In Shropshire in 1633, Enoch ap Evan, rumored to be an Anabaptist, decapitated his seventy-two-year-old mother and younger brother in “the impatience and rage of his fantastic spirit” in order “to vindicate the cause of God.”^43^ Contemporaries disagreed on whether or not he was insane.^44^ Religious zeal provided one explanation for why things had gone so badly wrong, but it did not mitigate his crime. It was, if anything, an aggravating factor, as such parricides were presented as earthly manifestations of rejection of the true God.

Clearly not all frenzies indicated insanity, temporary or otherwise. Some modern commentators have conflated categories that early modern people did not. In a 1624 ballad, William Purcas killed his mother in an alcohol-fueled rage, which was indeed depicted as a temporary loss of reason, but it was not, as some modern commentators have assumed, an example of temporary insanity.^45^ Drunkenness was not viewed as a form of lunacy, even though it could induce frenzied, violent, or otherwise unreasonable behavior. Robert Hicks’s lunatic rages were exacerbated but not caused by alcohol, as was mentioned above. Lunacy was not the consequence of self-indulgence and sin, whereas drunkenness was throughout the period viewed as an evil that could overcome both reason and human feeling.^46^ Violence fueled by alcohol was always the fault of the drinker. When Purcas lamented that drink had removed his reason and left him as “reasonless … as a brutish beast,” he had only himself to blame: the ballad begins with Purcas awaiting the death he deserved: “Ne’r villain did so vile a deed as I have done, I know.”^47^ Drunkenness did not mitigate murder. The most common form of homicide in which drunkenness played a part were those alehouse fights which resulted in verdicts of manslaughter—in these, both parties bore some responsibility for the violent encounter during which one had been killed. Those circumstances informed the legal distinction between murder (fully culpable homicide) and manslaughter (partially culpable homicide). Parricides were not conceptualized in the same way.

### The Selfish Child Who Lacked Compassion

The most common early modern explanation for parricide was that a selfish adult without compassion killed a parent who stood between him or her and something they desired: an inheritance, marriage, money, or freedom. Nowadays this is the least frequent explanation evoked for parricide. In the Purcas ballad, Purcas wished to live a lewd and profligate life of which his mother disapproved. He is presented not as insane nor even as hotheaded but rather as a coldhearted, vicious person who lacked human emotion and whose mother was a hindrance to the sort of life he desired.^48^ Henry Jones murdered his mother from “covetousness and unjust greedy desires after his Mother’s rightful Estate” of £100 in order to continue his “wild dissolute course of living.”^49^ A Cornishman cut his aged father’s throat for the inheritance he needed to fund his “youthful extravagancies.” He initially insisted that his father had committed suicide and then tried to frame his mother for the murder before confessing to having done it himself.^50^ A Hertfordshire man murdered his mother and tried to dispatch his father, too, because what they provided for him when he was “reduced to poverty by his irregular life” was insufficient, “the Youth having not liberty enough to indulge all his senses in lust.”^51^ In Sussex, another woman was murdered and the house robbed by a son who had squandered the estate his father had left him.^52^ Mrs. Palmer, a Worcestershire gentlewoman, was murdered by her son and three confederates for withholding an estate of £50 per annum.^53^ Elizabeth Cranbery poisoned her disabled and “ancient” stepfather, having grown “weary of the trouble” and inconvenience “of feeding him,” he having lost the use of his hands; the prosecution alleged at her trial that she was further motivated by him threatening, after an argument, to throw her out of the house.^54^

Many of these murders were committed apparently by those in their twenties and thirties, which reflects the practical and emotional contingencies of early modern family and household structure. Whereas the majority of young people spent their teens in the service of other households, most expected to marry and to establish their own households in their twenties. It is telling that many early modern parricides were inflicted upon aged fathers or widowed mothers who might literally be seen as barriers to an independent life or at least to one that was preferable to their current circumstances. This perhaps also goes some way to explaining why matricides were almost as common as patricides in early modern England and Wales.^55^ Of the forty-three parricides for which I have evidence, twenty-three (53.5 percent) of the victims were fathers and twenty (46.5 percent) were mothers of the perpetrators. The context of the household and family dynamics also contributed to early modern understandings of why sons outnumbered daughters as perpetrators of parricide by more than six to one.^56^ Not only did sons usually have more to gain financially than daughters when their parents died, but in a system that privileged primogeniture, widowed mothers in particular might be seen as a final obstacle to inheritances that otherwise seemed within reach. Nonlethal legal conflicts over resources and inheritances between widowed mothers and their sons were common enough.^57^ The great majority of familial tensions over such matters did not lead to homicide, of course. Nor were all sons who murdered parents likely to have been motivated by financial gain, especially when they used forms of violence that increased the chances of being caught, such as throat slitting. It is nonetheless striking that contemporaries explained most parricides in this way and depicted the parent–victim accordingly.

Accounts of parricide frequently evoked compassion for the murdered parent by portraying them in positive, sometimes ideal, terms. William Purcas’s mother feared that the “foul spotted fault” of drunkenness would bring her son to a wicked end, which, of course, is precisely what happened. She “gently” chided him, explaining that “this loathed crime” was a sin, and that one sin fed many others such as swearing and whoring, which in Purcas’s case, it did. Such maternal counsel chimed with advice in conduct literature: a mother “shall continually with most sweet maternal exhortations, move [her children] unto virtue, and stay them from vice; declaring unto them what a precious jewel virtue is … and contrariwise what a horrible monster sin is before the eyes of God.”^58^ Purcas’s response was all the more terrible therefore. His mother would “speak to me in love, / I’d answer her in rage, / Without all fear or reverence of title, or of age.” When finally, in a drunken fury, he drew his knife to shut her up, she fell to her knees before him and pleaded for her life:

And on her knees did beg,
that I her life would spare,
And 't were but for my soul, on which
she pray’d me have a care:
Oh spare me, son, she said,
forget not who I am,
Thy aged Mother do not then
thy ears against me dam.
Alas, how canst thou, son,
endure to see me kneel,
And beg and weep and wring my hands,
and no compassion feel?

The flowing of Purcas’s mother’s tears—a well-known trope of openheartedness and warmth—is contrasted with Purcas’s hardness and lack of compassion. This conceit is so important that it is immediately repeated, this time from Purcas’s perspective:

Thus kneeling would she beg,
and begging, weep apace;
And weeping, she would wring her hands,
in lamentable case.
Yet nothing was I mov’d,
with all her piteous moan,
My heart for her did feel no grief,
but was as hard as stone.

In the face of his mother begging and begging and weeping and weeping, William Purcas remained unmoved. He stabbed her to death. The words attributed to his mother and to Purcas emphasized her lack of culpability for his actions. I am unconvinced by the assertion that the language of this and other similar ballads “underlined [the sinners’] passivity”^59^; these ballads emphasized the active culpability of their protagonists in accordance with the execution ballad genre. The ballad ends with William eulogizing his mother, warning others to heed his example, and declaring that he deserved to be hanged 10,000 times over for what he had done.^60^

In these accounts of parricide, compassion is presented as the normal response to the mother’s suffering and distress. Purcas said that his story “will draw forth brinish tears from any that have human hearts.” The “ghastly spectacle of a murdered mother,” another trial pamphlet stated, would “touch” and “mollify” even “the obdurate heart of [a] wicked son.” Indeed, “who hears of a mother wilfully murther’d by her own son, but his senses startle, and his heart is instantly brim-full of horror and indignation?”^61^ Accounts of matricide frequently evoke the “monstrous” paradox of “tak[ing] away the life of her who gave you yours, that bare you in her womb, dandled you on her knees, and nursed you in her bosom.”^62^ In one execution ballad, the mother’s corpse seems to “speak” to the now remorseful son, in whose imagination it points to her hands, her breast, and her womb.^63^ The physical–emotional bond between mother and child could not be replicated for fathers. It was harder for patricide to be described with such pathos unless fathers were very old and frail. Nonetheless, similar rhetoric was adapted, only a hardened “wretch” could murder his own father and “stop the vital source from whence he first drew breath” in order “to indulge a vicious passion.”^64^

Throughout the period, tales of patricide most frequently emphasized, along with the child’s rejection of parental proper discipline and good counsel, the sheer brutality of the killing. In 1606, the Catholic recusant, Inigo Jeanes, murdered his father because the latter repeatedly tried to talk him out of his popish pursuits. When his father refused to permit mass to be celebrated in his house, Inigo bludgeoned him to death with a heavy “club or beetle (wherewith they used to cleave wood) … and struck him violently on the head to the ground.” To make doubly sure his victim was dead, Inigo then smashed his father’s back to pieces with an iron bar.^65^ This type of extreme violence marked out even spontaneous killings as fully culpable. In 1700, Henry Jackson murdered his “tender and loving mother, who always tendered his welfare equal with her own.” She had continued to treat him thus even after he had fallen in with “lewd company” and had become debauched in “such a degree of excess, that swearing, cursing, drunkenness, and the abominable sin of whoredom became familiar to him; wasting his estate in extravagancy and riot; till at last it came to a low ebb.” Jackson rejected his mother’s concerns “with scorns and reproaches, saying, he was now of age and knew what to do without the advice and counsel of a silly doting old woman.” When finally she ceased giving him money to spend on “his extravagancies,” he killed her. In a premeditated attack, Jackson came knocking at her door late one night and in “a submissive manner … told her it was her Prodigal return’d, who now had a true sense of his errors and failings, and was heartily sorry for the wicked courses he had taken, but most of all for offending her, in despising her good advice, and if now at last she would receive him, he would be a very obedient son.” His mother, “overjoyed,” rushed down and let him in, whereupon “this ungracious wretch … shut the door, knocked her down, and with his knife, pitiless and remorseless cut her throat, and having robbed the house, departed.” Like many parricides, and in line with the crime pamphlet genre, Henry’s initial coldheartedness and lack of remorse was replaced by penitence and sorrow by the time of his trial and execution, where Henry wept bitterly and claimed he was tormented by the sound of dear, aged mother’s “groans and cries always sounding in [his] ears.”^66^

In the typical early modern parricide “without compassion,” the murderer’s actions were distinguishable from those of lunatics by the fact that he or she was aware of his or her actions and knew he or she was wrong—an imperative if the reader is to identify with them in accordance with the conventions of the murder pamphlet and execution ballad. Nor were such killings depicted as hot-blooded “chance medley” homicides for which a manslaughter verdict might be returned, even when the immediate circumstance of the crime was a spontaneous response to a parent’s actions or words. In such cases, the parent’s behavior was not construed as having been unreasonable. Even brief reports which gave little or no details indicated this.^67^ In 1727, a young man in Somerset “took up an axe, and therewith he by several blows cleaved his mother’s skull, and as she lay weltering in her blood, he punch’d her with the other end [of the axe] till she expired,” all because, ostensibly, she had hesitated to bring him more ale, he having arrived home “much in liquor.”^68^ In 1740, a newspaper reported a sorry tale of a young man who lost his temper when his mother reproved him first for going to the alehouse instead of church and second for lying about it: in a rage, he stabbed her in the chest and back and then cut his father’s throat.^69^ The lack of empathy displayed by these killers was not presented as a pathological condition—a form of mental disorder that differentiated them from “normal” people—as it is in modern psychological assessments of parricide. Rather the lack of compassion and empathy was viewed as evidence of an internal weakness to which everyone might potentially succumb, a process made much more likely by the excessive consumption of alcohol, which was itself portrayed as a sinful trait.

The discourse of the sinner whose lesser sins gradually led to greater ones which in turn led to the gallows became prominent in the early seventeenth-century murder pamphlet arc of sin, divine Providence, and redemption, and was still in force in mid-eighteenth-century discussions of crime and punishment.^70^ Even short newspaper reports drew upon it. In 1722, readers were informed that an eighteen-year-old Flintshire girl had “a mind to dress more nicely than her father would allow,” and so broke open his chest and stole £5 (the equivalent of about £400 nowadays) in order to buy herself some fabulous clothes at Chester Fair “to make herself fine.” When these extraordinary purchases caused her father to suspect that it was she who had stolen his money, she poisoned him. At her father’s funeral, her brother told her he feared their father had been murdered; “to stop his mouth,” she poisoned him too. The local minister, however, also suspecting ill play, insisted that surgeons inspect her brother’s corpse before he buried him. Symptoms of arsenic poisoning were discovered, and upon evidence that the girl had purchased a sufficient quantity of arsenic at an apothecary’s in Holywell, she was apprehended. “This,” declared the *Weekly Journal or Saturday’s Post,* “is a powerful lesson against pride, which first influenced this wretched creature to commit theft, and then urged her on to parricide and murther.”^71^

Avarice, jealousy, impatience, self-indulgence, and lust—all of which might ultimately lead to murder—were understood to be common emotions. These horrible stories were shocking to an early modern audience. But they were also warnings about the brute that lies potentially within us all. The stories about parricide that circulated in popular print in the seventeenth and first half of the eighteenth centuries suggest that it was likely to emerge in situations where there were preexisting domestic tensions and in the immediate context of a spontaneous verbal dispute. These familiar scenarios served not to normalize parricide, which remained a hideous and ghastly crime, but to suggest that an act as terrible as the brutal murder of one’s own mother or father might be within anyone’s scope.^72^

### Parricide and Parental Fault

A frequent explanatory context given for parricide in recent decades is severe and ongoing parental abuse or neglect of children. This was not a common explanatory model in the seventeenth and eighteenth centuries. Early modern understandings of “abuse” and “neglect,” and the damage potentially caused by such treatment, were rarely evoked as explanations for what might drive a person to kill their parent. Yet a minority of cases, even when generally sympathetic to the parent’s plight, hinted that events might have been avoided had the child been raised differently. This narrative was less about abuse and cruelty than it was of indulgence, overfondness, and a lack of appropriate discipline. Nor did it exculpate the killer in any way: legal and societal concepts of provocation did not extend to situations beyond the immediate interaction of the parties.

The narrative of parental fault could have an additional explanatory role in tales where the main focus was the child’s ill living and cruelty. Things started going wrong for Henry Jackson, for instance, when following his father’s death, “his mother’s over-fondness recalled him home” from university, after which “he got acquainted with lewd company and grew very extravagant.”^73^ The roots of a triple murder by a fellow who poisoned his wife, father-in-law, and mother-in-law in order to gain all that was theirs and to marry his mistress was traced back to his father dying when he was young, which “was the cause of a too loose Education.”^74^ Mr. Charles John Drew, a Suffolk lawyer who died from multiple gunshot wounds, was described by the prosecution as “an indulgent Father.” Drew furnished his only son with £200 a year, which the boy spent on lewd and dissolute pursuits, a lifestyle which soon outstripped his allowance.^75^ Other authors who discussed the case went further. Drew junior had been “brought up in a boorish ignorance.” His parents having separated, “the Father never troubled his head about him, and his Mother was as regardless, so that he was bred up in a rough manner, and among persons in the lowest class of life.” Drew senior was “of a very strange perverse and most severe Temper”; in the years following his marital separation, he refused to speak to his children at the table and for hours on end. Some declared he was not indulgent but cruel. He “barbarously cut off” his son and was “a man of [such] an unhappy temper, and possessed of so much severity, that it even amounted to brutality.”^76^ None of several accounts of the case suggested that Drew junior should avoid the death penalty for the murder. But neither did they present the murdered parent favourably.^77^

Such pamphlets reveal the tension between acknowledging that children might feel legitimately angry toward parents on the one hand and the illegitimacy of any angry response of their own on the other. The Puritan, John Downame, wrote in 1616 that anger and the *desire* for revenge might be legitimate when it was stirred up on “weighty and necessary causes” such as sin. Sin should indeed make us angry. But the same applied to children and parents as to a poor man and his social superior: a poor man had no authority “to show his anger in the same manner … as he would to his equal or inferior” because “though he may justly be angry with his sin, yet he is to reverence his place and calling.”^78^ Similarly, a child “must not show his anger towards his father as the father showeth his towards his [child], for he is bound to fear and reverence his person though he justly hate his sin.” A child’s feelings of “violence and fury” toward a parent might thus be warranted but they had no business expressing or acting out those emotions. Even when “justly incensed by the barbarous tyranny of [one’s] merciless father,” one should do no more than “rising from the table and departing.”^79^ In letters ostensibly written by Elizabeth Jeffries as she awaited trial for the murder of her adoptive father, an uncle, Jeffries herself claimed that his “partial, and I am afraid fatal favour to me is the source of all my misfortunes.” Although he did not send her to school, “his Indulgence to me made up for everything else,” making her his primary heir, which “indulgence … was naturally look’d upon with an evil eye” by other relatives “who thought themselves altogether as deserving of his favour.”^80^ However, Jeffries’ case is unusual in that it presents sexual abuse as part of the context for parricide. The epistolary pamphlet ends not only with her admission of guilt—“having all along represented myself as an innocent person, when in my conscience I knew I was the most horrid hypocrite and monster of cruelty that ever existed”—but also with the confession that her uncle was a vulgar, brutal man with no religion or morals, who “took great pains to debauch” her “in which I blush to tell you he was too successful.” The awful truth was to be “a warning to parents or guardians how they principle those under their care, lest like my uncle they reap the fruit of their own wickedness.”^81^

Very rarely did an account of parricide suggest that a parent deserved their fate. Yet even this did not mitigate parricide. A ballad of 1600, reprinted in the 1650s—probably due to its renewed relevance after Charles I’s regicide—presented parricide as a direct consequence of a father’s cruelty toward his own parents. The ballad begins with an impoverished and infirm elderly couple walking 100 weary miles to their affluent son’s home to seek relief. Our hearts leap with theirs when they see the house in the distance. We respect their dignity as they trim their shoes and hose and put on clean neckbands to make themselves presentable. We feel their trepidation as “the woman with shaking head, the old man blind and lame” knock gently on the door, “fearing to offend.” We are moved by the woman’s heartfelt words: “This is thy father gentle son, / and I thy loving mother / That brought thee up most tenderly, / and lov’d thee above all other. / I bore thee in this womb, / these breasts did nourish thee. / And as it chanced I often danced thee on my tender knee.” And we are horrified by their son’s response: he turns them away, refuses to allow them even to sleep in his barn, and threatens to have them whipped as vagrants if they tarry. Through their tears, the couple lament their son’s “wicked,” “unkind,” and “cruel” deed, warning that the Lord may have as little pity for him at such time as he himself might stand in need. As a consequence, we have little care for the plight of this man when he becomes the victim of his own sons. For shortly afterward, his children having observed the way their father treated his parents “in their great wretchedness” as if they were worthless, decided to kill him for his land, silver, and gold. When he begged for his life, his children remained unmoved: “with Dagger and sword/they mangled him monstrously” and “buried him in a stinking ditch where no man could find him.” But the story did not end there. We are not invited to empathize with his children either when they are bludgeoned to death by a cousin who also had his eye on the gold. All ended how it should, when the cousin was arrested, tried, and executed for their murder. Thus, we see “God’s vengeance on them all … deservedly for their cruelty.”^82^ Viewed as the most extreme form of filial disobedience, parricide could be—and sometimes was—seen to arise partly as a consequence of parenting. In the early modern period, this did not excuse the killer, but it invited questions about how one should raise a child.

A further context for parricide was that of parents forcing their children to marry persons whom they did not love and/or preventing them from marrying the person whom they did. Parents of most social backgrounds were expected to be involved in the selection of their children’s spouse, and young people were certainly not to marry without parental consent. In practice, the relative parts played by parents and young people in choice of marriage partner varied considerably.^83^ Forced marriages as well as elopements were frowned upon, even among the aristocracy.^84^ Popular texts deplored the actions of tyrannical parents, fathers in particular, who privileged the economic benefits of their children’s marriage over their lasting happiness. Such accounts depicted marriage for love as the preferable alternative to greed for prestige or financial gain. A ballad of 1670 condemned a girl’s father for preventing her marriage to a poor farmer’s son by arranging for him to be press-ganged to sea. The resourceful young woman ran away to sea herself disguised as a surgeon. After fortuitously saving her sweetheart’s life after he was injured in battle, she revealed her true identity, and the couple returned home where, discovering that her father had conveniently died, they married and inherited his estate to the joy of the community.^85^ Sometimes the inflexibility of parents led to the unfortunately undesired spouse being murdered, in which case the victim was depicted as entirely innocent.^86^ When a parent was the murder victim, however, representing parricide became more complex, as is exemplified in the case of Mary Blandy.

## Portrait of a Parricide

Mary Blandy was tried at the Oxford Assizes in March 1752 for poisoning her father, Francis Blandy, a respectable attorney. The case produced a deluge of nearly forty pamphlets, scores of news reports, and several portraits in various media between the time of the murder and the trial. Mary was described as a good girl even by the lawyers who led the case against her: she was “genteel, agreeable, sprightly, sensible,” “a young Lady virtuously brought up, distinguished for her good behaviour and prudent conduct in life.”^87^ At the time of the tragedy, she was in her early thirties and unmarried. Her father had turned down several suitors whom he considered insufficiently affluent or well connected, and in order to gain the interest of suitable men, he had willfully spread a rumor that Mary’s dowry would be £10,000—a lie given that at his death his entire estate was valued at no more than £4,000. The promise of this dowry attracted Captain William Henry Cranstoun, an army officer who was a younger son of a Scottish peer. Mr. Blandy and his wife enthusiastically encouraged the courtship, Mary fell deeply in love, and Cranstoun eventually proposed. Mrs. Blandy was so sure of Cranstoun’s honor that she lent him money. However, matters took an unfortunate turn when it transpired, shortly after Mrs. Blandy premature (but nonsuspicious) death, that Cranstoun was a dishonorable cad who was clandestinely married to a woman in Scotland—from a known Jacobite family, no less—and the father of at least two bastards, including one got during his courtship of Mary. Mr. Blandy forbade the match.

In some accounts of what happened next, Mary is depicted as holding a naive or desperate belief in Cranstoun’s avowals that he was not married to the lady in question, a former mistress of his. He claimed rather that he had just pretended to be married to protect her reputation after she had given birth to one of his bastards. Other authors asserted that Mary by this time knew perfectly well that Cranstoun was a married man and that the couple had compacted to murder both impediments to their own union, she her father and he his wife.^88^ Whatever the case, when questioned by the coroner immediately after her father’s death, Mary maintained that she had not intentionally poisoned him. She confessed instead to having secretly administered a powder provided by Cranstoun, which he had claimed was a love philter to make Mr. Blandy once again well disposed and “kind to them in their affair.” She had no idea that the powders contained arsenic. It was Cranstoun who had administered the initial doses of powder in Mr. Blandy’s tea. When Mary later informed Cranstoun that she chose not “to give it to her father any more in tea as it would not mix well,” he immediately wrote back instructing her “to put it into some gruel or anything else that was thick,” which she did. A week later, her father was dead.^89^ Cranstoun escaped to France. Mary stood trial alone.

Numerous accounts constructed Mary Blandy’s parricide in familiar ways. In the officially sanctioned trial transcript, published with the Assize judges’ permission, the solicitor general, Henry Bathurst, opened the trial by emphasizing the treacherous nature of the offense: Mary had murdered a “father passionately fond of her.” She had “wickedly taken away his life, to whom she stands indebted for life,” and “deliberately destroyed, in his old age, him by whose care and tenderness she was protected in her helpless infancy.” The second crown prosecutor, Mr. Sergeant Hayward, articulated the motive: her father’s life had “become an obstacle” to Mary and Cranstoun’s affair, “and therefore he must be remov’d.” At the trial, Hayward directly addressed the many Oxford University students in attendance, lecturing them on “the dreadful consequences of disobedience to parents.”^90^ The manuscript prosecution brief went further:

she being a girl of very good sense, and [widely] read, … made herself agreeable to everybody, which greatly increased her father’s fondness, of which she took no small advantage, and at length gained the ascendant over her father, so far, as to do what she pleased, and to keep what company she pleased; and did not pay that due regard to his advice and instructions as a child ought to have done, which in all probability was the forerunner of the following catastrophe.^91^

Indeed, within a week of Mr. Blandy’s death, newspapers had already reported his daughter had murdered him because he had opposed her inclinations to keep company with Cranstoun: “in revenge for which, and *in order to be her own mistress*, she has perpetrated this unnatural crime of parricide.”^92^ These are familiar tropes in stories of parricide in which a coldhearted child without compassion for their parent murders in order to remove an obstacle to the life they wish to lead.

Yet at the same time, the terrible course of events was presented as having been set off by a fault in Mr. Blandy himself. Even the crown prosecutors pointed to Blandy’s “pious fraud” in “pretending he could give her £10,000 for her fortune …. But how short-sighted is human prudence! What was intended for her promotion proved his death and her destruction.”^93^ Some pamphlets described Blandy as “an indulgent father,”^94^ while others report his change of heart over Cranstoun as “the tyranny of her cruel father,” “what she termed the cruelty of her father.”^95^ None of these texts condoned murder, but they nonetheless created a narrative in which the murdered parent might not have been entirely blameless.

In 1752, descriptions of parricide thus continued to draw upon conventional discourses. But they also drew on newer ones, especially those of amatory fiction and the epistolary novel, which presented a different sort of sentiment and emotion and formed a competing set of narratives in which parricide might sit. One pamphlet contained what were allegedly letters exchanged between Mary and Cranstoun, while another declared itself to be written by her own hand and published at her dying wish, although this was met with a counterpublication that promised to explode “all the ridiculous and false assertations” of the other.^96^ Even the novelist and magistrate, Henry Fielding, opined that the cause of the entire affair was Mary’s “infatuation” with Cranstoun, which was “the only thing strong enough to overcome her otherwise high intelligence and goodness.”^97^ Another text contained a large number of letters said to be those exchanged by Mary and the aforementioned Elizabeth Jeffries who was convicted of parricide at the Essex Assizes around the same time. Here, both women were portrayed initially as victims—Mary having been “deluded and decoy’d by a worthless man” to become “the innocent cause of the death of a most dear and indulgent father,” and Elizabeth Jeffries entirely innocent of any involvement in her uncle’s death but destroyed by the envious and vengeful relatives who wished to prevent her from inheriting his fortune. But the story became increasingly whimsical as the women fantasized about setting up home together in a remote pastoral location after their hoped-for acquittals, before returning to harsh reality as first one and then the other was convicted and condemned to die. Although Jeffries confessed to having murdered her uncle, Mary retained, in this pamphlet, her innocence.^98^

Several newspapers conceded that “many contradictory reports [were] spread relating to Miss Blandy” within days of her arrest, leading at least one (in the minority) choosing “to omit saying anything about it” until they could be certain that what they reported was based on truth.^99^ By the time of the trial, public interest was intense and the array of “information” in circulation bewildering. Readers were “assured” that “Miss Blandy has desired not to be executed by a man, but a woman; and that she promised a woman five guineas and her clothes for doing the job.”^100^ One news report of the trial (later reiterated in a pamphlet) claimed that when Bathurst hinted that Cranstoun was attracted not to her but to her supposed dowry, Mary, who had remained unmoved when charged with a lack of humanity, “could not bear the least hint of want of beauty”: “the fire kindled in her eyes, and she discharged a look … full of such indignation and contempt, that it is inconceivable to any except those who beheld it.”^101^ After her conviction, there were rumors that she was to receive a pardon (she did not). A false report that the execution was scheduled for 3rd April resulted in vast crowds assembling at Oxford Castle gate three days early and waiting for many hours before “return[ing] home disappointed.”^102^ Accounts of the execution itself also diverged.^103^

**Figure 1.**
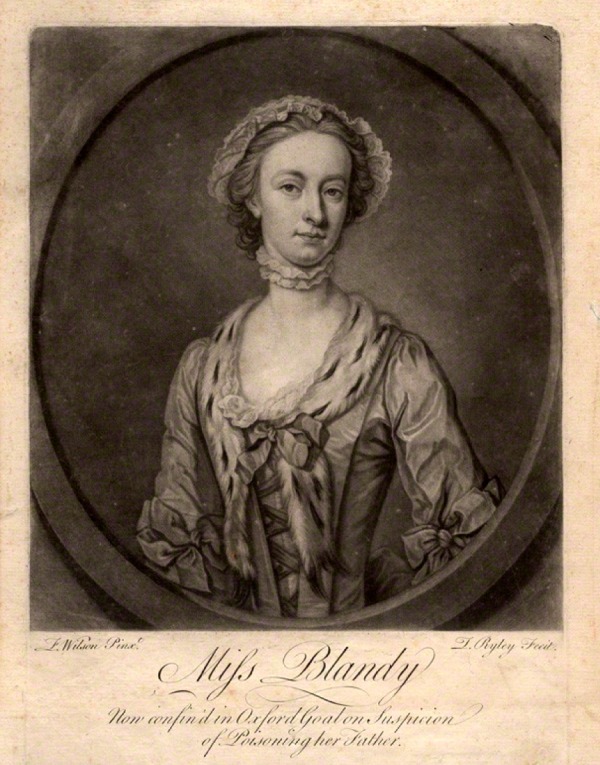
“Miss Blandy,” mezzotint by Thomas Ryley after F. Wilson. *Source*. ©National Portrait Gallery, London.

**Figure 2.**
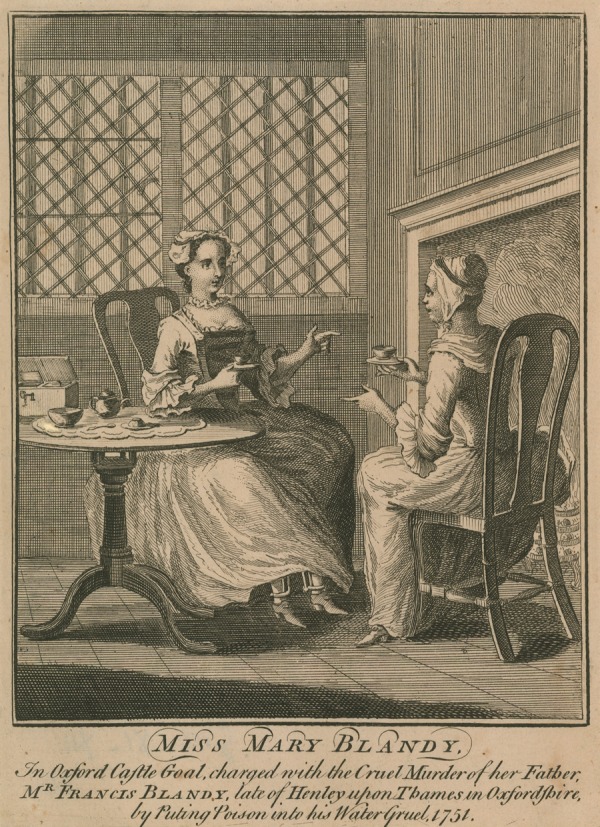
“Miss Mary Blandy, 1751”. Engraving. *Source*. ©Look and Learn/Peter Jackson Collection/Bridgeman Images.

**Figure 3.**
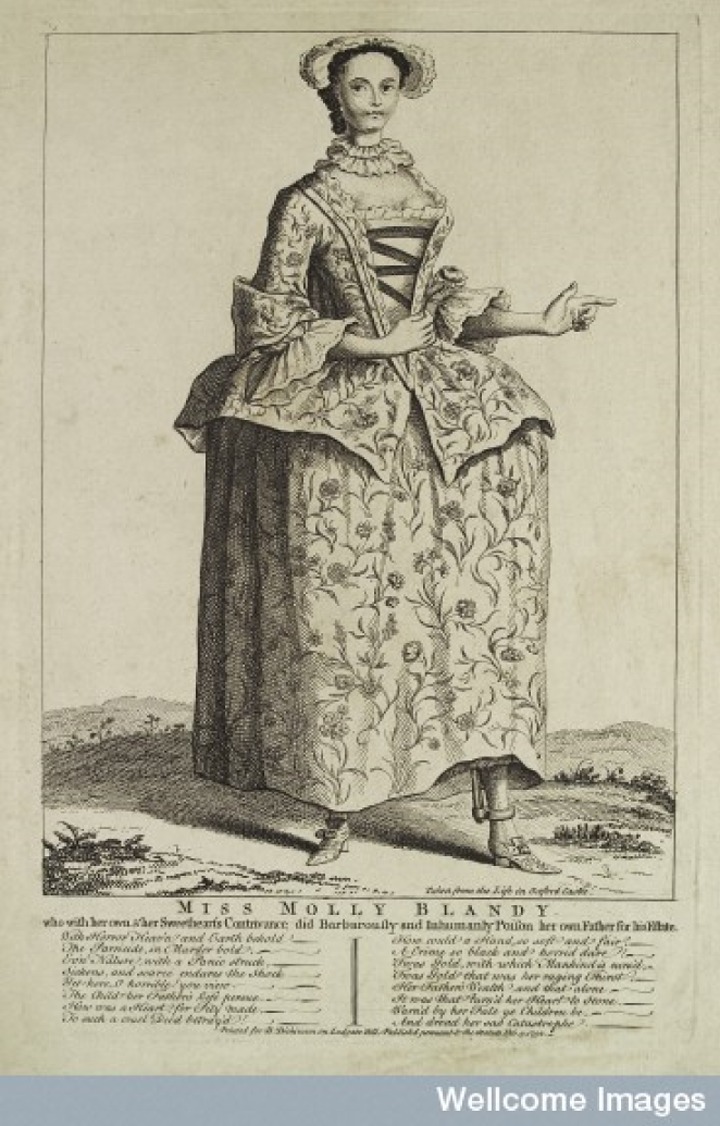
“Miss Molly Blandy,” printed for B. Dickinson, February 3, 1752. Etching. Wellcome Library, London.

**Figure 4.**
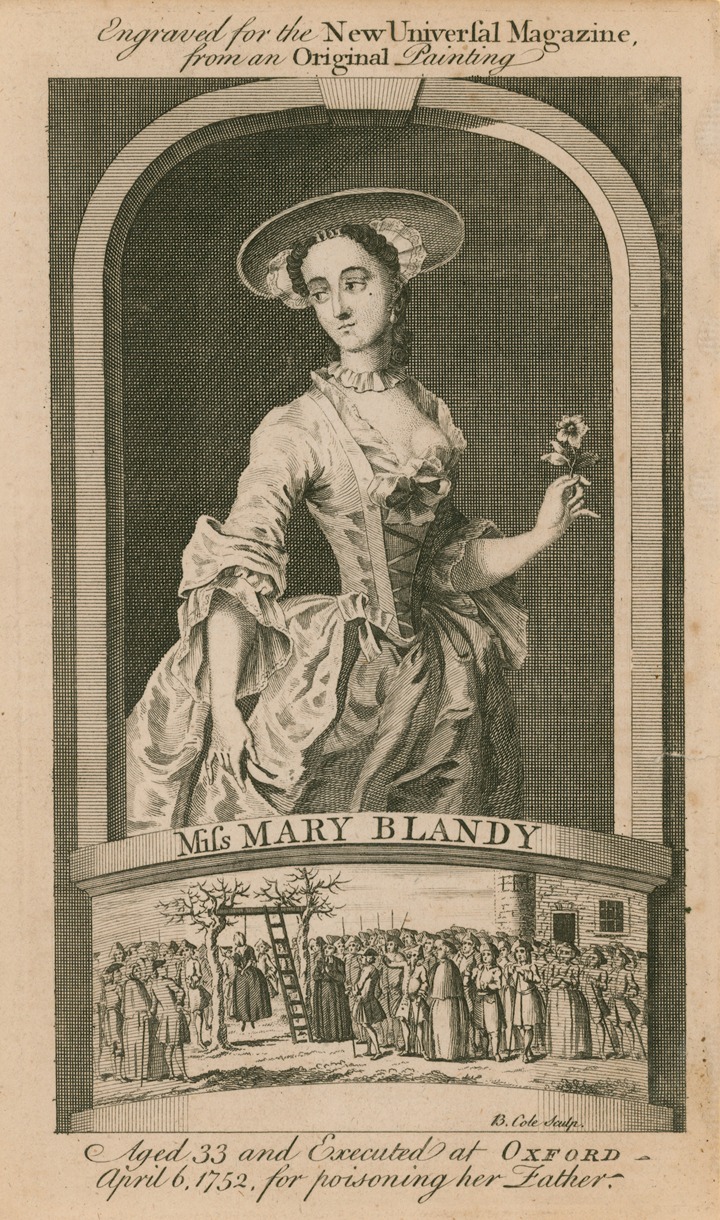
Portrait of Miss Mary Blandy engraved for *New Universal Magazine* from the original painting executed at Oxford on April 6, 1752, for poisoning her father. *Source*. ©Look and Learn/Peter Jackson Collection/Bridgeman Images.

Such tensions are neatly illustrated in the visual images of Mary in circulation before, during, and after her trial and execution. Figure 1 shows a charming mezzotint of Mary. The mezzotint was one of the most fashionable mass media in which portraits (usually copied from oil paintings for which the subject had sat) were disseminated, and which places her alongside a major means by which courtesans and actresses like Kitty Fisher and Fanny Murray were celebrated. Moreover, Blandy’s mezzotint was produced not only in the smallest (and cheapest) 6 × 4 inch format but was also available as a 14 × 10 inch print, which tells us that her image had a decent market. In Figure 2, Mary is taking tea with another lady. We might suppose her to be in her own parlor, but if we look closely we can see the bars on the windows and, under a slightly raised dress, that she is wearing leg irons; the lettering underneath informs us that Mary is her cell in Oxford Castle.^104^ Here she is again in Figure 3, looking ever so pretty in a nice frock in a pastoral scene. The contradiction is in the detail. The text informs us that the image is “Taken from life in Oxford Castle,” and again her gown does not cover her shackles. Both the presence of a maid and the ignominy of being fettered were matters Mary Blandy raised in her own defense during her trial and were central to several pamphlets discussing her case.^105^ The inscription reads “Miss Molly Blandy who with her own and her sweetheart’s contrivance did barbarously and inhumanly poison her own father for his estate.” And—just in case the observer has not kept up with current affairs—there is an accompanying moral in verse. But the verse underneath provides an unexpected motive: it does not mention Cranstoun or marriage, instead recalling the most common parricide narrative of the coldhearted child killing their parent for money, “How could a hand so soft and fair” commit “a crime so black and horrid?” The answer, “‘Twas gold, with which mankind is curs’d, / ‘twas gold that was her raging thirst/Her father’s wealth and that alone/it was that turn’d her heart to stone.” The verse ends by warning other children to take heed of her “sad catastrophe.” The catastrophe itself was depicted visually elsewhere, as in Figure 4, where the main image shows Mary looking whimsical and pretty, with her gallows scene underneath. As any eighteenth-century person knew, hanging was not a glamorous death.

It is ironic that the evidence that allows us to analyze Mary Blandy’s trial and reactions to it so fully is that of which she most complained. At her trial, Mary spoke out against the “hardships” she had endured as a consequence of rumors and published reports. She particularly resented the publication of “papers and depositions, which ought not to have been published, in order to represent me as the most abandoned of my sex, and to prejudice the world against me.”^106^ Solicitor General, Bathurst, acknowledged her feelings of violation at such media intrusion. He confirmed that “the printing what was given in evidence before the Coroner, drawing odious comparisons between her and former parricides, and spreading scandalous reports in regard to her manner of demeaning herself in prison, was a shameful behaviour towards her, and a gross offence against public justice.” The judge, summing up the case, said much the same.^107^ But these matters were immaterial. The jury were instructed to “disregard what you have heard out of this place.” The matter that they were to determine was whether when Mary gave the poison to her father she knew it to be poison and the effect it would have. The jury retired only for about five minutes before returning with their verdict: Mary Blandy was guilty. She was hanged on April 6, 1752.^108^

This article has explored the ways in which parricide was comprehended in England and Wales in the seventeenth and first half of the eighteenth centuries. We have seen that while interpretative early modern categories seem to chime in certain respects with modern ones, there are also significant differences. Parricide is commonly understood and explained in the present in terms of mental illness and parental abuse of their children. In the early modern period, both lunacy and the cruelty of parents were understood to be possible contexts in which parricide might arise, but neither were common. The dominant explanation was the gratuitous violence of a selfish individual who viewed the parent as an obstacle to be removed, and who acted without compassion. While this might seem similar to the modern pathologically violent offender who lacks empathy, the two differ in important respects. What is now seen as a mental disorder was then considered to be a state into which any normal individual might fall, should they not guard against sin. This remained the dominant discourse in which parricide (like other homicides and serious crime) was discussed at least until the mid-eighteenth century. However, other types of crime narrative emerged in the eighteenth century as popular trial accounts began to reflect broader cultural shifts that were reflected, too, in philosophy, aesthetics, and literature. Although conventional trial narratives made truth claims based on personal observation and individual detail, we see in the eighteenth century, a greater emphasis on the individuality rather than the universality of persons about whom stories were told. The widely publicized Mary Blandy trial demonstrates that while those conventional ways of making sense of parricide remained in force, parricide could be harnessed by authors to tell different sorts of stories that led the reader in alternative directions. Those routes, however, will have to be further explored elsewhere.

## Notes

1. Spelling in quotations from primary sources has been modernized, and capitalization and punctuation have sometimes been modified for clarity and consistency.

2. Conyers Place, *A Sermon Preached at Dorchester in the County of Dorset, January the 30th 1701/2* (London, UK: Printed and sold by J. Nutt, 1702), 5.

3. Sir Roger L’Estrange, *The Bloody Sons Warning-piece. Or, News from Reading in Berkshire* (London, UK: Printed for Thomas Johnson, 1676).

4. *The Tryal of Mary Blandy, Spinster: For the Murder of Her Father, Francis Blandy, Gent., At the Assizes Held at Oxford [on 29 February 1752]* (London, UK: Printed for John and James Rivington … in St Paul’s Church-yard, 1752), 3.

5. Philippe Ariès, *L’Enfant et la vie familiale sous l’ancien règime* (Paris, France: Plon, 1960); Philippe Ariès, *Centuries of Childhood: A Social History of Family Life* (New York: Knopf, 1962); Edward Shorter, *The Making of the Modern Family* (New York: Basic Books, 1975); Lawrence Stone, *The Family, Sex and Marriage in England, 1500–1800* (London, UK: Weidenfeld and Nicolson, 1977). Cf. Linda A. Pollock, *Forgotten Children: Parent–child Relations from 1500 to 1900* (Cambridge, UK: Cambridge University Press, 1983); Linda Pollock, *A Lasting Relationship: Parents and Children over Three Centuries* (London, UK: Fourth Estate, 1986); Hugh Cunningham, *Children and Childhood in Western Society since 1500* (Harlow, UK: Pearson Education, 2005).

6. Joanne Bailey, *Parenting in England, 1760–1830: Emotion, Identity, and Generation* (Oxford, UK: Oxford University Press, 2012); Claudia Jarzebowski and Thomas Max Safley, eds., *Childhood and Emotion: Across Cultures 1450–1800* (London, UK: Routledge, 2014).

7. Frances E. Dolan, *Dangerous Familiars: Representations of Domestic Crime in England, 1550–1700* (Ithaca, NY: Cornell University Press, 1994); Laura Gowing, *Domestic Dangers: Women, Words, and Sex in Early Modern London* (Oxford, UK: Oxford University Press, 1996).

8. George Closse, *The Parricide Papist, or Cut-throate Catholicke* (London, UK: for Christopher Hunt, 1606), 4.

9. *Tryal of Mary Blandy*, 3.

10. Forty-three parricides were discussed in scores of news reports and eighty trial pamphlets, ballads, and manuscripts relating to English and Welsh trials. *Proceedings of the Old Bailey* are given as *OBP*, with session date, defendant’s name, and trial reference number from *Old Bailey Proceedings Online* (www.oldbaileyonline.org, version 7.1, April 2013).

11. For these sources, see Dolan, *Dangerous Familiars*, 1994; Robert B. Shoemaker, “The Old Bailey Proceedings and the Representation of Crime and Criminal Justice in Eighteenth-century London,” *Journal of British Studies* 47, no. 3 (2008): 559–80; Michael Harris, *London Newspapers in the Age of Walpole: A Study of the Origins of the Modern English Press* (Toronto, Canada: Associated University Presses, 1987); Garthine Walker, *Crime, Gender and Social Order in Early Modern England* (Cambridge, UK: Cambridge University Press, 2003).

12. Sir William Blackstone, *Commentaries on the Laws of England*, vol. 4 (Oxford, UK: Clarendon Press, 1769), 202–3; Thomas Wood, *A New Institute of the Imperial or Civil Law* (London, UK: Printed by W. B. for Richard Sare …, 1704), 272–73, 353; J. B., *A Compendious Collection of the Laws of England, Touching Matters Criminal* (London, UK: Printed for John Bellinger … and Tho[mas] Dring, 1676), 7–8. Under Roman law, parricide originally applied to killing one’s child and one’s parent.

13. For example, *Daily Journal*, June 17, 1728.

14. Sir Edward Coke, *The Third Part of the Institutes of the Laws of England* (London, UK: Richard Atkyns and Edward Atkyns, 1670), 20; Blackstone, *Commentaries*, 202–3. See also Thomas Wood, *An Institute of the Laws of England*, vol. 2 (London, UK: Printed … for Richard Sare, 1720), 598; Sir George Mackenzie, *The Works of That Eminent and Learned Lawyer, Sir George Mackenzie of Rosehaugh, Advocate to King Charles II and King James VII*, vol. 2 (Edinburgh, UK: James Watson, 1722), 111.

15. Ferdinando Pulton, *De Pace Regis et Regni, Viz. A Treatise Declaring Which Be the Great and General Offences of the Realme, and the Chiefe Impediments of the Peace of the King and Kingdome* (London, UK: Printed … for the Companie of Stationers, 1609), fol. 111v.

16. Coke acknowledged that “some say that parricide was petit treason by the common law”: *Third Part of the Institutes*, 20.

17. John Walthoe, *The Common and Statute Law of England, Concerning Trials in High-treason, Misprision of Treason, and in All Other Crimes and Offences Relating to the Crown* (London, UK: Printed by J. Nutt …, 1710), 363.

18. Michael Dalton, *Country Justice* (London, UK: W. Rawlins and S. Roycroft, 1705), 338; Sir Matthew Hale, *Historia Placitorum Coronae: The History of the Pleas of the Crown*, vol. 1 (London, UK: Printed by E. and R. Nutt and R. Gosling … for F. Gyles …, T. Woodward …, and C. Davis …, 1736), 378. A number of parricides might thus be hidden in indictments for petty treason by servants.

19. Pulton, *De Pace Regis et Regni*, fols. 111–111v. The pertinent statute was 25 Edward III, c.5, Treason Act (1350).

20. Sir Edward Hyde East, *A Treatise of the Pleas of the Crown* (London, UK: J. Butterworth, 1803), 336.

21. *General Evening Post*, May 29–31, 1735.

22. *Country Journal or The Craftsman*, June 7, 1735.

23. *The Bloody Murtherer, Or, The Unnatural Son His Just Condemnation at the Assizes Held at Monmouth, March 8, 1671/2* (London, UK: Printed by H. Lloyd for Jonathan Edwin, 1672).

24. *Fog’s Weekly Journal*, June 7, 1735; *Weekly Miscellany*, June 7, 1735; William Hughes (matricide) and Elton Lewis (parricide-related murder of the aunt with whom he lodged): *OBP*, May 1735, Hughes, t17350522-4, and Lewis, t17350522-5. Both cases were discussed at length in a number of newspapers.

25. Closse, *Parricide Papist*, 4; *Weekly Journal or British Gazetteer*, February 4, 1721; *Pasquin Extraordinary*, March 11, 1723. See also *Weekly Journal or Saturday’s Post*, February 20, 1725.

26. For legal categories of homicide, see Walker, *Crime, Gender and Social Order*, 114–16.

27. Ibid., 113–58.

28. Kathleen M. Heide, *Understanding Parricide: When Sons and Daughters Kill Parents* (Oxford, UK: Oxford University Press, 2013), 14–15.

29. For reasons why these categories might be inappropriate even for nineteenth-century America, see Phillip C. Shon, *Respect, Defense, and Self-Identity: Profiling Parricide in Nineteenth-century America, 1852–1899* (New York: Peter Lang, 2014), 2–3.

30. Richard Moran, “The Origin of Insanity as a Special Verdict: The Trial for Treason of James Hadfield (1800),” *Law & Society Review* 19, no. 3 (1985): 31–42; Richard Moran, *Knowing Right from Wrong: The Insanity Defense of Daniel McNaughtan* (New York: The Free Press, 1981).

31. Thomas Young, *England’s Bane: Or, the Description of Drunkenness* (London, UK: Printed by William Jones …, 1617), sigs. B2–C2, at B2; Dalton, *Country Justice*, 351.

32. Dalton, *Country Justice*, 351; Roy Porter, *Mind-forg’d Manacles: History of Madness in England from the Restoration to the Regency* (London, UK: Athlone Press, 1987), 114–17.

33. Dana Y. Rabin, *Identity, Crime and Legal Responsibility in Eighteenth-century England* (Basingstoke, UK: Palgrave Macmillan, 2004).

34. Nigel Walker, *Crime and Insanity in England: The Historical Perspective*, vol. 1, 2 vols. (Edinburgh, UK: Edinburgh University Press, 1968); Joel Peter Eigen, “‘I Answer as a Physician:’ Opinion as Fact in Pre-McNaughtan Insanity Trials,” in *Legal Medicine in History*, ed. Michael Clark and Catherine Crawford (Cambridge, UK: Cambridge University Press, 1994), 167–99.

35. *Old England*, August 26, 1749.

36. *Daily Post*, July 23, 1740; *London Evening Post*, July 22–24, 1740; *Universal Spectator and Weekly Journal*, July 26, 1740; *London and Country Journal*, July 31, 1740. See also *London Journal*, June 19, 1725; *London Packet or New Lloyd’s Evening Post*, September 23–26, 1796.

37. Heide, *Understanding Parricide*, 10–14, passim.

38. *London Journal*, June 19, 1725; *Brice’s Weekly Journal*, June 25, 1725; *Newcastle Courant*, July 24, 1725.

39. Quoting Michael MacDonald, *Mystical Bedlam: Madness, Anxiety and Healing in Seventeenth-century England* (Cambridge, UK: Cambridge University Press, 1983), 117, 126–28.

40. MacDonald, *Mystical Bedlam*, 132–33.

41. *OBP*, April 1722, Robert Hicks, t17220404-52; the fuller version of the trial was published in *Select Trials … At the Sessions-house in the Old-Bailey … 1720 to 1724 Inclusive*, vol. 1 (London, UK: Printed for J. Wilford *…*, 1735), 155–56.

42. Closse, *Parricide Papist*, 8.

43. Peter Studley, *The Looking-glasse of Schisme Wherein by a Briefe and True Narration of the Execrable Murders, Done by Enoch Ap Evan, a Downe-Right Separatist, on the Bodies of His Mother and Brother …* (London, UK: Printed by R[ichard] B[adger] for Thomas Alchorne *…*, 1634), 29, 35, 38–42.

44. Richard More, *A True Relation of the Murders Committed in the Parish of Clunne in the County of Salop by Enoch ap Evan upon the Bodies of His Mother and Brother* (London, UK: Printed by T.B. for P. Stephens and C. Meredith *…*, 1641), 10–11, 19; Peter Lake, “Puritanism, Arminianism and a Shropshire Axe-murder,” *Midland History* 15, no. 1 (1990): 37–64; Alexandra Walsham, “Prophecy, Sorcery, Insanity, and the Elizabethan Puritan Movement,” *The Historical Journal* 41, no. 1 (1998): 27–66.

45. Joy Wiltenburg, “Madness and Society in the Street Ballads of Early Modern England,” *Journal of Popular Culture* 21 (1988): 108.

46. Young, *England’s Bane*, passim.

47. *The Wofull Lamentation of William Purcas, Who for Murtherin[g] His Mother at Thaxted in Essex Was Executed at Chelmsford. To the Tune of the Rich Merchant* (London, UK: Printed for Francis Coules, dwelling in the Old-Baily, 1624).

48. *Wofull Lamentation of William Purcas*, 1624.

49. *The Bloody Murtherer, Or, The Unnatural Son*, 62.

50. William Reddish, *Strange News from Stratton in Cornwal: Or, A True Relation of a Cruel B[l]oody Murther* (London, UK: Printed for I. Coniers *…*, 1680).

51. *Post Boy*, March 18–20, 1697.

52. *The Unnatural Son, or, A Sad and Deplorable Relation of the Unfortunate End of H. Jackson at Horsham Near Sussex* (London, UK: Printed for R. Brown, 1700).

53. *Post Boy*, November 29 to December 2, 1707.

54. *Weekly Journal or Saturday’s Post*, April 23, 1720; *Evening Post*, April 26–28, 1720; *OBP*, April 1720, Elizabeth Cranbery, t17200427-43; *Weekly Journal or British Gazetteer*, May 7, 1720.

55. Work on the nineteenth and twentieth centuries that is based on a wide variety of parricide offenders (rather than being focused only on adolescent offenders or on adults who have been referred for forensic mental health examinations) reveals similar patterns. See Phillip C. H. Shon, “Sources of Conflict between Parents and their Offspring in Nineteenth-century American Parricides: An Archival Exploration,” *Journal of Forensic Psychology Practice* 9, no. 4 (2009): 249–79; Shon, *Respect, Defense, and Self-identity*, 2014; Dominique Bourget, Pierre Gagné, and Mary-Eve Labelle, “Parricide: A Comparative Study of Matricide Versus Patricide,” *Journal of the American Academy of Psychiatry and the Law* 35, no. 3 (2007): 306–12; Amanda Holt and Phillip C. H. Shon, “Exploring Fatal and Non-fatal Violence Against Parents: Challenging the Orthodoxy of Abused Adolescent Perpetrators,” unpublished paper, 2015. I am very grateful to Dr. Holt and Professor Shon for sharing their unpublished research with me.

56. In my sample, thirty-two males (84.2 percent) and six females (15.8 percent) were accused of parricide. For the gendered nature of early modern homicide, see Walker, *Crime, Gender and Social Order*, 113–58.

57. Tim Stretton, “Widows at Law in Tudor and Stuart England,” in *Widowhood in Medieval and Early Modern Europe*, ed. Sandra Cavallo and Lyndan Warner (London, UK: Routledge, 1999), 193–208; Garthine Walker, “Keeping it in the Family: Crime and the Early Modern Household,” in *The Family in Early Modern England*, ed. Helen Berry and Elizabeth Foyster (Cambridge, UK: Cambridge University Press, 2007), 67–95.

58. Thomas Becon, *The Worckes of Thomas Becon Whiche He Hath Hitherto Made and Published* (London, UK: Imprinted by John Day *…*, 1564), fos. 678r–78v.

59. Wiltenburg, “Madness and Society,” 108.

60. *Wofull Lamentation of William Purcas*, 1624.

61. *The Bloody Murtherer, Or, The Unnatural Son*, 9, 2–3.

62. Ibid., 13–14.

63. *The Penitent Sonnes Teares for His Murdered Mother by Nathaniel Tyndale* (London, UK: s.n., 1624).

64. *The Suffolk Parricide, Being, the Trial, Life, Transactions, and Last Dying Words, of Charles Drew, of Long-Melford, in the County of Suffolk* (London, UK: Printed for J. Standen *…*, 1740), 3.

65. Closse, *Parricide Papist*, 7–8.

66. *The Unnatural Son, or, A Sad and Deplorable Relation of the Unfortunate End of H. Jackson at Horsham Near Sussex*, 7, 2–4.

67. For example, *Country Journal or the Craftsman*, May 3, 1729; *Universal Spectator and Weekly Journal*, May 3, 1729; *Fog’s Weekly Journal*, May 3, 1729.

68. *Daily Journal*, August 16, 1727; *British Journal*, August 19, 1727; *Weekly Journal or British Gazetteer*, August 19, 1727.

69. *Universal Spectator and Weekly Journal*, December 27, 1740.

70. See, for instance, newspaper coverage of the trial of John Dell in *Domestick Intelligence or News Both from City and Country*, December 16, 1679, and for the trial, *OBP*, t16791210-10; William Reddish, *Strange News from Stratton in Cornwal*, 1680; *A True Relation of a Barbarous Bloody Murther, Committed by Philip Standsfield upon the Person of Sir James Standsfield His Father* (London, UK: Printed for J. Conyers *…*, 1688); *Post Boy*, November 29 to December 2, 1707. Peter Lake, “Deeds against Nature: Cheap Print, Protestantism and Murder in Early Seventeenth-century England,” in *Culture and Politics in Early Stuart England*, ed. Kevin Sharpe and Peter Lake (Standford, CA: Stanford University Press, 1993), 257–84; Peter Linebaugh, “The Ordinary of Newgate and His Account,” in *Crime in England 1550–1800*, ed. J. S. Cockburn (London, UK: Methuen, 1977), 246–69; Andrea McKenzie, *Tyburn’s Martyrs: Execution in England, 1675–1775* (London, UK: Hambledon Continuum, 2007).

71. *Weekly Journal or Saturday’s Post*, December 2, 1721; *Weekly Journal or British Gazetteer*, December 2, 1721.

72. This perhaps began to change in the eighteenth and through the nineteenth centuries, when parricide became pathologized with other forms of extreme violence. J. A. Sharpe, “‘Last Dying Speeches:’ Religion, Ideology and Public Execution in Seventeenth-century England,” *Past & Present* 107 (1985): 144–67; Peter Lake and Michael Questier, “Agency, Appropriation and Rhetoric under the Gallows: Puritans, Romanists and the State in Early Modern England,” *Past & Present* 153 (1996): 64–107; Garthine Walker, “Everyman or a Monster? The Rapist in Early Modern England, c.1600–1750,” *History Workshop Journal* 76 (2013): 5–31.

73. *The Unnatural Son, or, A Sad and Deplorable Relation*, 2.

74. *Three Inhumane Murthers, Committed by One Bloudy Person, upon His Father, His Mother, and His Wife, at Cank in Staffordshire* (London, UK: Printed for H. E., 1675), 1.

75. *The Genuine Trial of Charles Drew, for the Murder of His Own Father, at the Assizes … for the County of Suffolk*, 2nd ed. (London, UK: Printed for C. Corbett, 1740), 8–12.

76. *Suffolk Parricide*, 4–5.

77. See also *The Unnatural Son. Being the Whole Tryal and Condemnation of Charles Drew, of Long Melford in Suffolk, Esq* (London, UK: s.n., 1740). For unpublished material on the case, see The National Archives (hereafter TNA): PRO C205/15/10 Chancery: Petty Bag Office: Special Commissions of Inquiry, Murder and Felonies, Charles Drew otherwise Thomas Roberts, late of Long Melford [Suffolk], gent for the murder of his father.

78. John Downame, *Spiritual Physicke to Cure the Diseases of the Soule* (London, UK: Gabriel Simson for William Iones *…*, 1616), fos. 8v–10r, 14v.

79. Ibid., fos. 16v, 14v, 17r.

80. *Genuine Letters that Pass’d between Miss Blandy and Miss Jeffries, Before and After Conviction* (London, UK: Printed for J. Scott *…*, 1752), 7–8.

81. *Genuine Letters that Pass’d between Miss Blandy and Miss Jeffries*, 28, 31.

82. *A Most Excellent New Ballad, of an Olde Man and His Wife Which in Their Olde Age and Misery Sought to Their Owne Children for Succour, by Whom They Were Disdained & Scornfully Sent Away Succourlesse, and How the Vengeancc [sic] of God Was Justly Shewed Upon Them for the Same* (London, UK: Printed for W.B., 1600). Another imprint of the same ballad was printed for F. Coles, T. Vere, and W. Gilbertson between 1654 and 1664.

83. Catherine Frances, “Making Marriages in Early Modern England: Rethinking the Role of Family and Friends,” in *The Marital Economy in Scandinavia and Britain, 1400–1900*, ed. Maria Ågren and Amy Louise Erickson (Aldershot, UK: Ashgate, 2005), 39–56; Naomi Tadmoor, *Family and Friends in Eighteenth Century England* (Cambridge, UK: Cambridge University Press, 2000); Amanda Vickery, *The Gentleman’s Daughter: Women’s Lives in Georgian England* (New Haven, CT: Yale University Press, 1998), esp. ch. 2.

84. Alan Mcfarlane, *Marriage and Love in England: Modes of Reproduction, 1300–1840* (Oxford, UK: Blackwell, 1986), 134.

85. *The Valiant Virgin; or, Phillip and Mary: In a Description of a Young Gentlewoman of Worcestershire (a Rich Gentlemans Daughter) Being in Love with a Farmers Son, Which Her Father Despiseing, Because He Was Poore, Caus’d Him to be Prest to Sea* (London, UK: Printed for Thomas Passenger *…*, c.1670).

86. For example, Thomas Deloney, *The Lamentation of Master Pages Wife of Plimmouth* (London, UK: s.n., 1635).

87. *Tryal of Mary Blandy*, 4, 9; *The Genuine Speech of the Hon. Mr [Bathurst] at the Late Trial of Miss Blandy* (London, UK: Printed for J. Roberts in Warwick-Lane, 1752), 7. These follow closely the manuscript evidence for the case: for example, TNA: PRO Treasury Solicitor’s Papers TS/11/864, 2948, various examinations and depositions and TNA: PRO ASSI 5/72/10 (Oxford Assizes 1752), Mary Blandy to Cranstoun, letter stopped at the Post House, August 1, 1751.

88. *Genuine Speech of the Hon. Mr [Bathurst]*, editor’s note, 9.

89. TS/11/864, 2948, Examination of Mary Blandy taken before the coroner, August 16, 1751; TNA: PRO State Papers Scotland Series II (1688–1783), SP 54/41 f. 137, Examination of Mary Blandy, August 16, 1751.

90. *Tryal of Mary Blandy*, 4, 9–10; *Genuine Speech of the Hon. Mr [Bathurst]*, 6.

91. TS/11/864, 2948, Prosecution brief, “The King against Mary Blandy.”

92. *Whitehall Evening Post or London Intelligencer*, August 17, 1751; *London Daily Advertiser and Literary Gazette*, August 17, 1751; *London Evening Post*, August 17, 1751; my italic.

93. *Tryal of Mary Blandy*, 4; *Genuine Speech of the Hon. Mr [Bathurst]*, 7.

94. *A Genuine Account of the Most Horrid Parricide Committed by Mary Blandy, Spinster, upon the Body of Her Father Mr. Francis Blandy, Gent* (Oxford, UK: Printed for and sold by C. Goddard *…*, 1751), 4.

95. *An Authentick Narrative of That Most Horrid Parracide [sic] Committed on the Body of Mr. Blandy*, 2nd ed. (London, UK: Printed for M. Cooper *…*, 1752), 11.

96. *Original Letters to and from Miss Blandy and C- C- …* (London, UK: Printed for S. Johnson …, 1752); *Miss Mary Blandy’s Own Account of the Affair between Her and Mr Cranstoun* (London, UK: Printed for A. Millar *…*, 1752); *A Candid Appeal to the Publick, Concerning the Case of the Late Miss Mary Blandy* (London, UK: Printed for J. Gifford *…*, 1752).

97. Henry Fielding, *Examples of the Interposition of Providence in the Detection and Punishment of Murder* (London, UK: Printed for A. Miller *…*, 1752), 87.

98. *Genuine Letters that Pass’d between Miss Blandy and Miss Jeffries*, 16, 14, 18–22.

99. *Whitehall Evening Post or London Intelligencer*, August 20, 1751.

100. *Daily Advertiser*, March 17, 1752.

101. *Covent-Garden Journal*, March 28, 1752; *Genuine Speech of the Hon. Mr [Bathurst]*, 1752.

102. *London Daily Advertiser*, April 4, 1752; *London Evening Post*, April 4, 1752.

103. For example, *London Daily Advertiser*, April 7, 1752; *Read’s Weekly Journal or British Gazetteer*, April 11, 1752.

104. This engraving is a nineteenth-century copy of the original illustration which appeared in *A Genuine Account*, frontispiece.

105. *Tryal of Mary Blandy*, 31; *The Genuine Trial at Large of Mary Blandy, Spinster, for Poisoning Her Late Father Francis Blandy, Gent* (Belfast, Northern Ireland: Printed by James Magee *…*, 1752), 32–33; *A Candid Appeal to the Publick*.

106. *Tryal of Mary Blandy*, 31. Words to the same effect were attributed to her in *Genuine Trial at Large of Mary Blandy*, 32–33. Specific grievances such as that reports that she planned to escape (and thus had to be kept in leg irons) can be traced in the newspapers, for example, *General Evening Post*, August 17, 1751.

107. *Tryal of Mary Blandy*, 35.

108. Ibid., 45.

